# What the functions of consciousness are depends on what one thinks consciousness is

**DOI:** 10.1098/rstb.2024.0311

**Published:** 2025-11-13

**Authors:** Joseph E. LeDoux

**Affiliations:** ^1^ Professor Emeritus: Center for Neural Science, New York University, New York, NY 10003, USA

**Keywords:** consciousness, animal consciousness, brain evolution, memory and consciousness, functions of consciousnss

## Abstract

The word ‘consciousness’ is often used as if it is a single thing, and as if everyone knows, in a general sense, what that thing is. The very notion of a theory of consciousness implies that someday this thing will be accounted for. But suppose that multiple kinds of consciousness exist. If so, an adequate theory of consciousness would have to be multifaceted rather than unitary. And, accordingly, an account of the function or functions of consciousness would depend on the kind or kinds of consciousness one is referring to. Herein, I use a tripartite taxonomy of human consciousness and explore the relation of each kind to its underlying pre-conscious cognitive processes and their neural underpinnings. I also consider how each kind of consciousness may have evolved, and what the adaptive functions of each may have been.

This article is part of the theme issue ‘Evolutionary functions of consciousness’.

## 1. Introduction

If you do not know what you are looking for, you’ll never find it. It therefore helps to have a clear conception of what you are looking for before you start seeking it. Because we have been tasked here to consider the evolutionary functions of consciousness, it is crucial that we start by considering what consciousness refers to.

One broad distinction is between the condition of being alive, awake and responsive to the world, as opposed to being aware of one’s mental states. These are sometimes referred to as creature and mental state consciousness [1]. Creature consciousness exists in all animals, but knowing which animals might have mental state consciousness is considerably more difficult to ascertain. This article is about mental state consciousness.

## 2. Evolution of mental state consciousness

Human bodies differ in unmistakable ways from the bodies of other animals. Being part of the human body, the human brain differs in important ways from the brains of our primate ancestors, and theirs from their mammalian ancestors (Fuster 2020; [2–7]). It should, therefore, not be controversial to suggest that the mental states experienced by different animals, if they have them, may be different from what we possess, in kind and/or in degree. If this is the case, then the evolutionary functions of consciousness would also likely be different in different animals. This does not imply that humans are ‘exceptional’. It simply means that all species are, by definition, different.

A useful starting point is that evolution often builds on what exists [8]. Hence, it is unlikely that human mental state consciousness arose out of the blue. Instead, it likely evolved by building on what our mammalian ancestors possessed. A key question, then, is whether these ancestors possessed mental state consciousness itself, or instead just the non-conscious cognitive underpinnings that underlie human mental state consciousness. Addressing this question requires some way of scientifically assessing the conscious capacities of non-human mammals. Without that, it is not possible to understand the function or functions of consciousness in them.

## 3. The scientific problem of animal consciousness

Much has been written in support of animal consciousness (e.g. [9–20] ). But others have expressed scepticism about such claims (e.g. [21–28]). The controversy is less about whether non-human animals are conscious than about what counts as scientific evidence for consciousness in them.

The history of consciousness research in psychology provides a useful lens on this topic. Psychology arose as an experimental science in the late nineteenth century and took two forms [29]. One was a human psychology that used introspection to study consciousness. The other branch was comparative (i.e. animal) psychology, which was greatly influenced by Darwin’s proposal of a continuity of mind (consciousness) across humans and our animal ancestors [28].

Georges Romanes, an acolyte of Darwin, and a pioneer of comparative psychology in the UK, was a leader in the animal consciousness movement. He said that an animal’s behaviour is an ambassador of its mind [30]. Edward Thorndike, an early animal psychology pioneer in the USA, was also influenced by Darwin. He pioneered research on voluntary behaviours acquired by stimulus–response associative learning. Just as Darwin proposed biological selection at the level of the species, Thorndike proposed behavioural selection at the level of the individual organism [31]. Thorndike’s famous ‘law of effect’ held that subjective (i.e. conscious) feelings of pleasure and pain stamped in the stimulus–response associations. Though he later recanted this subjective account, it was too late, as consciousness had become the default explanation of complex behaviour in animals.

In pursuing animal consciousness, comparative psychologists used their own introspections to intuit consciousness in animal behaviour. The rule of thumb was, ‘an animal has consciousness when it exhibits the kind of behaviour that is characteristically conscious in human beings’ [29,32]. This strategy came to be known as ‘analogy with human behaviour’ [22] and was applied widely to animals, and even to single-cell protozoa [33].

Judging what an animal is experiencing on the basis of what we experience is perfectly fine as a way of interacting with pets but is not sufficient as a scientific criterion. This is in part what led John Watson to call for an objective science of behaviour in animals and humans in which subjective mental states of consciousness had no place [34,35]. As is well known, the movement had legs, and consciousness was removed from the menu in psychological research and theory for decades.

Today, with behaviourism largely (though not completely) out of the picture, consciousness is a thriving area of research in humans and animals. But much like in the late nineteenth century, analogy with human behaviour is still a leading approach in research on animal consciousness. And it is still problematic.

For example, when rats are in danger, their blood pressure and heart rate rise, and they ‘freeze’ or flee. Because humans often feel afraid when in danger, it is assumed the feeling of fear is what causes the behavioural and physiological responses. But mounting evidence shows the circuits that control the objective responses are not the circuits that construct the subjective feeling of fear [36–39]. Specifically, the circuits underlying the conscious experience of fear operate in parallel with the reflex-like behavioural and physiological control circuits that operate non-consciously. As a result, even if rats feel some form of ‘rat fear’, the responses measured are mere correlates of consciousness. This does not mean that there is no conscious control of behaviour in humans and other mammals. It just means that if you use overt behaviour to measure consciousness, you have to use responses that depend on conscious control. This is not circular reasoning since the dissociation can be made in humans. But it is not so easy to make the dissociation in non-human animals, which is why analogy with human behaviour is so often called upon. Because behaviour is all we can measure in animals, we should be judicious in choosing which behaviours to call upon, since not all behaviours are equal as ways of assessing consciousness, even in humans [27].

Some may be persuaded that studies showing that local anaesthetics relieve pain-like behaviours in animals is evidence for human-like experiences of pain in animals. But observations of instinctive and reflexive recuperative responses to tissue injury do not prove that the animal is consciously experiencing pain. It may well be experiencing pain, but such behaviours are controlled non-consciously and are not suitable as indicators of human or animal consciousness. Behaviours that depend on cognition are considerably better than reflexes and instincts as measures of consciousness. This is why Jonathan Birch, a leading animal consciousness researcher, proposed that understanding of ‘consciousness-linked cognitive abilities’ might be the way to go [12]. But cognition does not equal consciousness since not all cognitions are conscious [40].

The best measure of consciousness is verbal self-report [41–44]. For example, people can respond verbally or non-verbally to something they are conscious of but can only respond non-verbally to something they are not conscious of. This is why verbal report, though not perfect, is so useful in distinguishing conscious from non-conscious control of behaviour. And because all responses are non-verbal in non-human organisms, there is no built-in means that can tell you which, if any, non-verbal behaviours reflect conscious mental states, and which do not. This does not mean that human-like language is necessary to be conscious. It just means that, without language, mental state consciousness is difficult to verify scientifically. At the end of the day, claims of consciousness in animals necessarily rest more on intuitions and beliefs than on data.

I agree that we should err on the side of caution when making ethical decisions, but that does not mean that we should loosen the scientific criteria when trying to demonstrate animal consciousness. As Marian Stamp Dawkins has argued, because of the difficulties in scientifically demonstrating animal consciousness, tying the problem of animal welfare to the problem of animal consciousness hinders rather than helps animal welfare [45].

Why are we on any firmer ground studying consciousness in humans than in animals? Barring a brain disorder, all humans have brains with the same basic structural components and functional capacities. Given that, if I am conscious of my mental states, I can with some confidence assume that you have this capacity as well. Like any species trait, consciousness varies across individuals, but will be present, or at least is potentially present, in all members of our species. The further away a species is from us, the harder it gets to make the case that they have human-like conscious states.

## 4. Partitioning human consciousness

It might be helpful in matters of consciousness to distinguish different kinds of mental state awareness. For example, the psychologist Endel Tulving proposed a three-way partition of human consciousness: *autonoetic* (explicit self-awareness of one’s existence over time), *noetic* (explicit awareness of facts and concepts about the world or oneself) and *anoetic* (implicit/tacit awareness of one’s self and the world in the moment [46–48]).

While consciousness is hard to study, even in humans, Tulving’s scheme simplifies the task by associating each kind of consciousness with a distinct kind of empirically measurable memory: autonoetic consciousness with episodic memory; noetic consciousness with semantic and conceptual memory; and anoetic consciousness with procedural memory. Furthermore, he also associated each kind of consciousness with a distinct kind of knowledge: autonoetic consciousness with self-knowledge; noetic consciousness with factual knowledge; and anoetic consciousness with tacit (non-knowing) knowledge. Janet Metcalfe and Lisa Son went further, associating each with different kinds of metacognition [49].

Autonoesis and noesis are well defined kinds of conscious experiences, which each have a straight-forward relation to well defined kinds of explicit memory. But the relation of anoesis to implicit procedural memory is more complicated [27].

Procedural memory is a grab-bag notion defined by being non-conscious. That is, it is any kind of memory that is not consciously experienced—i.e. that is not episodic or semantic memory. Furthermore, while episodic and semantic memory are each associated with circumscribed, mostly cortical, brain circuits, the many kinds of procedural memory are widely spread throughout the brain and include learning capacities of sensory and motor cortex, habit learning in the basal ganglia, Pavlovian threat conditioning in the amygdala, and Pavlovian eyeblink conditioning in the cerebellum, among others.

Here is the rub. How can anoesis be a kind of consciousness if it is based on unconscious learning and memory? The key to the conundrum is that anoesis is not unconscious [27]. To understand this, we have to unpack Tulving’s bewildering notion of ‘non-knowing knowledge’. For starters, we can think of it as tacit rather than explicit experience and it can also be thought of in relation to what William James referred to as the ‘halo’, ‘fringe’ or ‘penumbra’ of ‘consciousness’ [50]. For example, following James, the philosopher Bruce Mangan argued that fringe states come with a tacit feeling of rightness, i.e. feelings of warmth and intimacy, that lack explicit content but imbue higher cognitive states of consciousness with a feeling of familiarity [51,52]. Lacking explicit content, these make minimal demands on working memory and articulation resources and are ever present. The social scientist Asher Koriat makes a similar point. He refers to such states as ‘sheer subjective experiences’ that are shaped by unconscious processes and that lack explicit content—think of *gut feelings* and *tip-of-the-tongue* phenomena [53]. These feelings allow you to know that your body and mental states are yours without you having to explicitly affirm this [16–18,24,27,36,54,55]. Anoetic states, in short, endow noetic and autonoetic consciousness with tacit feelings of warmth and familiarity.

The beauty of Tulving’s scheme for understanding human consciousness is obvious. Because each kind of mental state consciousness is associated with a kind of memory, each kind of consciousness has a non-conscious anchor in the human brain. The brain areas and circuits involved in episodic and semantic memory are well understood, but the neural basis of anoesis has not been studied much and is a ripe area for research.

There are currently two hypotheses about the brain basis of anoesis. Panksepp and Vandekerckhove argued that primitive subcortical circuits are a sufficient neural foundation of anoesis [17,18]. By contrast, my view is that anoesis depends on re-representation of lower-order subcortical states in medial areas of cortex.

In effect, Tulving’s model can be seen as a variant of the higher-order theory of consciousness since non-conscious lower-order states are transformed into higher-order conscious experiences [56]. Tulving emphasized specific phenomenological experiences that accompany episodic, semantic and procedural remembering. But as noted above, distinct kinds of meta-cognition underlie Tulving’s different phenomenal experiences [57]. In my multi-state, hierarchical framework, a complex network of neural cascades transforms pre-conscious cognitive states, including meta-cognitive states, into autonoetic, noetic and anoetic conscious experiences [21,27,36,58].

## 5. Why does memory matter to consciousness?

What, you might ask, is the importance of Tulving’s partition for understanding consciousness in general. The fact is, meaning comes from memory. We do not innately know about letters and numbers, nor about cats, trees, mountains, pencils, tuna salad sandwiches or cars. We must learn what these, and other, common things are. And once we do, we use the memories we form to recognize the objects later as members of a particular category, and not members of other categories. This is hardly a novel point. The nineteenth-century psychologist and physiologist Hermann von Helmholtz argued that past experiences, stored as memories, allow us to draw ‘unconscious conclusions’ about what is present [59]. His contemporary, the sensory physiologist Ewald Hering [60], wrote: ‘Memory connects innumerable single phenomena into a whole, and just as the body would be scattered like dust in countless atoms if the attraction of matter did not hold it together so consciousness—without the connecting power of memory—would fall apart in as many fragments as it contains moments’ (Hering, 1870). In the 1930s, the British psychologist Fredric Bartlett introduced the idea that memories are the basis organized knowledge structures called *schema* that we use to make sense of the world around us [61]. Meaningful conscious experiences require memory.

## 6. How can we know what kinds of consciousness other animals might possess?

Let us start with a paper by Karl Lashley in 1950. It was one of the key factors that helped bring the mind back to psychology after the long reign of behaviourism [62]. Lashley proposed that every conscious thought is preceded by non-conscious cognitive processing. Another way of saying this is that pre-conscious processing can, but does not necessarily, become conscious. This semi-behaviourist twist allowed psychologists to study the mind without necessarily calling upon consciousness as an explanation.

The way I like to think about Lashley’s idea is that, in order for a conscious experience to occur, the border between pre-conscious cognition and consciousness has to be crossed. Measurable meta-cognitive processes that are each pre-conscious to a different kind of conscious experience take us to the border, but not necessarily across.

If we could achieve detailed understanding of Tulving’s three kinds of consciousness [63], and their representation in the human brain, that would offer a way to glean what kind or kinds of consciousness other animals *might*, and *might not*, possess, given the ways their brains are like, and different from, ours [24,27,56,64].

Whether other primates and other mammals actually have the capacity to cross the border between pre-conscious processing and conscious experience cannot be known with certainty. Evidence that they possess the pre-cognitive processes and circuits underlying the different kinds of consciousness would be a significant achievement, not a mere consolation prize. But I have an empirical hypothesis about how to assess whether non-human primates and non-primate mammals *might* cross the border.

Tacit anoetic consciousness may have evolved in early mammals by the expansion of the reptilian pallium into the medial wall of the mammalian hemispheres, including the hippocampal formation and medial prefrontal cortex, areas that are shared by all mammals. If research on humans corroborates the role of these medial cortical areas and circuits in anoesis in humans, it would suggest that all mammals might have the capacity for anoetic consciousness, since all mammals have medial prefrontal cortical areas.

With the evolution of primates, the frontal lobe expanded beyond medial cortex to form the anterior part of the lateral neocortex [2–5,7,65,66]. All primates possess similar lateral prefrontal areas, including the dorso-lateral prefrontal cortex, which integrates sensory and memory information in working memory [5,65,67]. In humans, these areas have been implicated in conscious awareness of objects and events in the external world (noetic consciousness). Given the similarity of lateral prefrontal cortex in human and non-human primates, the latter may have non-verbal noetic consciousness.

The prefrontal cortex expanded significantly again with the evolution of humans and the other great apes. Particularly important is a region of the anterior frontal cortex called the lateral frontal pole. It is lacking in other primates and is involved in complex mental processes unique to humans, such as flexible multitasking and hierarchical, recursive inferential reasoning [68–74]. The lateral frontal pole has also been specifically implicated in episodic cognition [75] and autonoetic consciousness [76]. Further research on the contribution of the lateral frontal pole to autonoesis is needed.

I make no effort to take this approach beyond mammals since the relation to human traits diminishes as the evolutionary distance from humans increases. And since human consciousness is the only kind of consciousness we truly know exists, the basis for speculating about human-like consciousness in animals that are not in our evolutionary past, such as protostome invertebrates, is tenuous at best. Calling upon parallel evolution of consciousness in such cases is also tenuous since, unlike body parts, there are no physical traits to analogize with. There is of course behaviour, but as I have noted, analogy with human behaviour is problematic as a measure of consciousness, and the problems multiply when speculating beyond mammalian ancestors [27].

## 7. Sentience and anoesis

The simplest definition of sentience is the sheer ability to respond to sensory stimuli. A paper by Merker is often cited as evidence for sentience in humans and other animals [77]. But in using primitive behavioural responsiveness to sensory stimuli as the criterion, Merker only showed evidence of creature consciousness. Most who use the term ‘sentience’ argue for phenomenal feelings (qualia). But this cannot be demonstrated by simply measuring sensory-triggered behaviours, or even cognitive control of behaviour.

Marie Vandekerckhove offered an interesting suggestion—that sentience falls between anoesis and creature consciousness [17]. If so, sentience could be something more than being awake and alive, but less than anoetic consciousness. But this requires some way to distinguish sentience from anoesis above and creature consciousness below, which might be challenging even in humans [27].

Issues related to animal consciousness are subtle and complex and require semantic and conceptual clarity and interpretive restraint. Speculations about sentience and other forms of consciousness in non-human animals should be more openly acknowledged as speculations, rather than treated as facts that are so obvious, so intuitively correct, that they must be true. Some may say that my intuitions about animal consciousness in this article are no better than those commonly used to identify consciousness in non-human animals. The difference is that I openly acknowledge my intuitions as speculations, rather than as facts that are so obvious they must be true.

## 8. The functions of anoetic, noetic and autonoetic consciousness across mammals

What might be the functional advantages of mental state consciousness? The most general answer is that consciousness opens up novel forms of decision-making and behavioural control [27,78]. But let us be more specific and address this for autonoesis, noesis and anoesis.

In early mammals, expansion of the pallium in the medial walls of the hemispheres may have allowed tacit anoetic feelings of rightness to enhance behavioural flexibility in survival activities such as foraging, avoiding danger and selecting mates, as compared with the rigidness of reflexive and instinctual reactions in the vertebrate ancestors of mammals.

With lateral neocortical cortical expansion in primates, additional survival advantages related to behavioural control may have involved the use of explicit cognitive (i.e. mental) models based on semantic and conceptual memory to predict the consequences of actions. These may have enabled noetic consciousness in primates and allowed them to have explicit conscious semantic and conceptual content. The older mammalian capacity for anoesis, in turn, provides tacit phenomenal feelings associated with the explicit content of noetic consciousness in primates.

With additional expansions of neocortex and older medial cortex, and the emergence of language and verbal-based semantic, conceptual and episodic memory, cognitive noesis and autonoesis may have made it possible for humans to know themselves verbally and non-verbally as entities with a past and present, and also to anticipate possible futures, with anoesis providing the phenomenal feel, the warmth and intimacy of these explicit conscious states.

Regardless of whether states of consciousness can actually be experienced in non-human mammals, the evidence described shows the kinds of cognitive and neural underpinnings that could serve as pre-conscious states could take non-primate mammals to the consciousness finishing line for anoetic consciousness and non-human primates to the noetic consciousness finishing line. That itself would say a lot about their cognitive capacities and their propensity for consciousness and would be a significant milestone that should not be viewed as a mere consolation prize. For example, consider how far bird episodic cognition went by using the term ‘episodic-like’ memory to avoid the unprovable claim that birds possess autonoetic consciousness [79].

These ideas are, of course, highly speculative. But by linking the speculations to facts about the structure and functions of the human brain and following the evolutionary history of the relevant circuits underlying different kinds of conscious experiences in humans back through mammalian history, we would have some empirical grounding for speculations about consciousness in non-human mammals, and about the evolutionary functions of anoetic, noetic and autonoetic consciousness. That said, what we would end up with would be an understanding of cognitive underpinnings of consciousness, not evidence for mental state consciousness itself. But I believe that would qualify as significant scientific progress.

## Data Availability

This article has no additional data.
